# A missing vas deferens: practical implications for urologists performing vasectomies and managing infertile men

**DOI:** 10.1590/S1677-5538.IBJU.2016.05.03

**Published:** 2016

**Affiliations:** 1 ANDROFERT, Andrology & Human Reproduction Clinic, Campinas, SP, Brasil

The vas deferens derives from the Wolffian (mesonephric) duct and shares a common origin with the kidney (1). Intrinsic Wolffian duct defects may result in failure of the vas deferens to develop, a condition that can occur in isolation or combined with renal agenesis or malformations. A missing vas, that is, unilateral absence of vas deferens, albeit relatively uncommon may be found by urologists performing vasectomies or evaluating men with fertility problems (1, 2). It is, therefore, important that urologists be aware of the practical implications when finding such a case scenario.

In this issue of the International Braz J Urol, Miller and colleagues give us useful guidance by reporting the prevalence of congenital unilateral absence of vas deferens in a cohort of 23,013 men presenting for vasectomy over a 20-year period in Quebec City, Canada (3). Among the confirmed cases, namely, those with i. No prior genital surgery or trauma and ii. A missing vas at the time of vasectomy, and iii. Confirmed sterility by a post-vasectomy semen analysis (PVSA) after unilateral vasectomy, a missing vas was found once in every 479 men subjected to vasectomy. In their study, most vasectomies had been performed using a non-scalpel technique combining thermal cautery and fascial interposition (4), which has shown to be as effective as ligation and fascial interposition (5). To our knowledge, the paper by Miller et al. is the largest series reported to date thus making it sound to assume that most urologists performing vasectomies will find such a case in their career. Interestingly, the authors identified a group of 34 men (0.15%) in whom a missing vas was suspected but could not be confirmed, mostly due to the absence of a PVSA to confirm sterility or a history of prior surgery or scrotal anomaly that might account for the missing vas.

The study of Miller and colleagues highlights three relevant aspects for practicing urologists that need to be discussed further. First, the importance of physical examination before the vasectomy and during fertility evaluation. Second, the role of post-vasectomy semen analyses, and lastly, the importance of surgical planning in difficult vasectomy cases.

Palpation of the vas deferens is essential and should be included as part of the routine physical examination in all men seeking vasectomy or fertility (6). The finding of a missing vas should prompt urologists to order an abdominal ultrasound study to detect any renal anomalies (6). In healthy men seeking vasectomy, it might be argued that ultrasound is unnecessary as the finding of a single kidney is not clinically relevant. An objection is that although up to 80% of men with a congenital unilateral absence of vas deferens (CUAVD) have ipsilateral renal agenesis, the kidney may be present and other renal malformations such as ectopia, malrotation, fusion or polycystic disease may occur (7). Despite not warranting further intervention, informing the affected men that they have only one kidney or a renal anomaly is good medical practice as it may prompt such men to take a better life-style, thus preventing the occurrence of type 2 diabetes and hypertension that may impact their renal function later in life (8). On the other hand, 1–2% of males investigated for infertility have congenital absence of vas agenesis (1, 6, 9). Whereas the condition is often associated with bilateral vas absence and azoospermia and most men with CUAVD are fertile, men with a single vas may present with mild oligozoospermia as testicular sperm output is cut in half (10). Furthermore, CUAVD may be associated with mutations in the cystic fibrosis transmembrane conductance regulator (CFTR) gene (1). The most alarming fact is that if both the male and female partners carry such mutations- a carrier frequency of 4% has been reported in Caucasian women- the newborn may present with congenital bilateral absence of vas deferens (CBAVD) and therefore infertility due to obstructive azoospermia or with the life-threatening autosomal disease named cystic fibrosis (1, 6, 11). Having said that, proper counseling seems advisable to all men with CUAVD regardless of their fertility status. In a man seeking vasectomy and therefore presumably fertile there is a small possibility that his male offspring harbor CBAVD if his female partner is a carrier of CFTR mutations. Identification of such condition earlier in life may be important not only for diagnosing infertility in the offspring but also for counseling about the chances of paternity and the risks associated with the use of assisted reproductive technology (ART) (11, 12). And in those individuals with fertility problems, particularly if candidates for ART, genetic screening of CFTR mutations is not a mere academic exercise; it is deemed necessary to allow couples to make informed decisions about their plan of parenthood (1, 12, 13).

A post-vasectomy semen analysis is recommended to all men subjected to vasectomy. In recent guidelines issued conjointly by the British Andrology Society and the British Association of Urological Surgeons, assessment of one semen specimen preferably examined within 1 hour of the collection in a laboratory that uses proper methods- is enough to confirm sterility if no sperm are seen (14). PVSA should take place a minimum of 12 weeks after vasectomy and after a minimum of 20 ejaculations (recommendation grade B). The reason is the earlier the testing, the higher the chance of a false-positive result. And data indicates that by 20 ejaculates, 80% of men show azoospermia or sperm numbers below detectable levels (15). Men with fewer than 3 ejaculations per week reach azoospermia approximately 5 weeks later than those with a higher ejaculation frequency (16). This means that not only the interval between vasectomy and PVSA is important but also the number of ejaculations. According to the aforementioned guidelines, in the presence of residual sperm vasectomy success is confirmed if <100000/mL non-motile sperm. However, this “special clearance” with non-motile spermatozoa is still under discussion (17, 18), as proper laboratory methods are crucial to ensure accuracy of results (19, 20). Interestingly, in the study of Miller et al., approximately 30% of all men subjected to vasectomy failed to comply with the recommendation of providing a specimen for analysis. The rate of non-compliance dropped to 20% among those in whom a unilateral vasectomy had been performed -probably due to the perception of the uncertainty of its success- but the figures were still high. Since surgeons are ultimately responsible for counseling their patients about potential risks and complications, including vasectomy failure and recanalization, it seems crucial to emphasize patients the importance of the PVSA, both verbally and written.

Lastly, given the higher rate of misdiagnosis reported in men with prior scrotal surgeries or testis abnormalities, it must be admitted, as suggested by Miller et al., that men with a suspected absent vas ipsilateral to such abnormalities would be better treated where a vasectomy can be combined with testis delivery. In our center, our preference is to perform vasectomies on an outpatient basis under intravenous sedation with propofol in association with spermatic cord block with Marcaine (21). If needed, the testis can be delivered and the scrotal contents explored without jeopardizing the effectiveness of the surgical intervention.

## CONCLUSIONS

A missing vas (unilateral congenital absence of vas deferens) is found once in approximately 480 men subjected to vasectomy. Palpation of the vas deferens is essential and should be included as part of the routine physical examination in all men seeking vasectomy or fertility. The finding of a missing vas may be associated with renal agenesis / abnormalities and or genetic mutations with potential risks for the offspring. Patients should be offered proper counseling and further testing whenever indicated. A post-vasectomy semen analysis is recommended to all men subjected to vasectomy. PVSA should take place a minimum of 12 weeks after vasectomy and after a minimum of 20 ejaculations. Evaluation of a single semen specimen is enough to confirm sterility if no sperm are seen. Men with a history of prior surgery or scrotal abnormalities in whom a missing vas is suspected may require scrotal exploration to avoid misdiagnosing the absent vas.
